# Social participation of patients with epidermolysis bullosa: barriers and facilitators in education, employment, and economic life - a biographic interview study

**DOI:** 10.1186/s13023-026-04329-y

**Published:** 2026-03-25

**Authors:** Vinzenz Hübl, Lotta Berninger, Britta Dawal, Cristina Has

**Affiliations:** 1 Department of Dermatology, Medical Center, Faculty of Medicine, University of Freiburg, Freiburg, Germany; 2 Department of Educational-and Social Sciences, University of Applied Sciences Südwestfalen, Soest, Germany

**Keywords:** Social participation, Epidermolysis bullosa, Barriers, Facilitators, Environmental factors, ICF

## Abstract

**Background:**

Epidermolysis Bullosa (EB) is a rare, inherited skin disorder characterized by skin fragility, leading to painful blisters and wounds. While protective measures are essential for managing EB, they can inadvertently restrict physical activity and social participation. Recognizing this dilemma, the current psychosocial care guidelines for EB emphasize the need to support social participation as a key priority. Accordingly, this study explores barriers and facilitators influencing participation in education, employment, and economic life, as conceptualized by the International Classification of Functioning, Disability, and Health (ICF).

**Methods:**

A qualitative research design using biographical interviews was employed. Twelve adults with EB were recruited via purposive sampling. Interviews were transcribed and analysed through deductive qualitative content analysis, guided by the ICF framework.

**Results:**

During the early stages of education, peer attitudes and bullying were identified as major barriers, while parental advocacy, supportive teachers, and peers helped mitigate exclusion. In the later stages of education (higher education and vocational training), frequent transitions in teachers, classrooms, and peers required participants to continuously renegotiate adjustments, which before used to be a one-time task. In employment, barriers included discrimination during job applications and inadequate support from employment agencies, whereas flexible arrangements (e.g., remote work) promoted participation. In economic life, complex bureaucratic procedures hindered access to social security benefits and medical reimbursements, increasing participants’ financial and psychological burden. Patient organizations significantly alleviated these burdens by assisting with paperwork and administrative navigation.

**Conclusions:**

Living with EB necessitates constant care to avoid physical stressors, yet protective measures can unintentionally restrict participation. This study identifies 17 environmental factors shaping participation across education, employment, and economic life, with uninformed attitudes emerging as a persistent barrier in all life areas. Supporting patient organizations in their advocacy efforts may help reduce stigma, improve awareness, and ultimately enhance social participation of individuals with EB.

**Supplementary Information:**

The online version contains supplementary material available at 10.1186/s13023-026-04329-y.

## Background

Epidermolysis bullosa (EB) is a rare, inherited skin disorder characterized by skin fragility. Minimal mechanical stressors cause painful blisters and wounds [1]. EB is classified into four major types: EB simplex (EBS), junctional EB (JEB), dystrophic EB (DEB), and Kindler EB (KEB). Over 30 subtypes are distinguished, each with distinct molecular and clinical manifestations [2]. Disease severity can range from mild, localized to severe, disabling, and life-limiting subtypes [1].

Currently, no cure exists for EB [3]. Disease management focuses on controlling symptoms and preventing complications, requiring meticulous wound care, and minimizing mechanical stress [4]. Consequently, daily activities must be planned in terms of the potential risks they pose [5]. This generates a dilemma: the necessary measures to protect the skin can inadvertently limit physical activity and participation in social life [6]. Striking a balance between caution and participation is crucial, as excessive protection can impact developmental processes and well-being [7–9]. Beyond the need for caution, physical limitations, such as restricted mobility and impaired hand function, can limit participation in everyday activities [6]. The visibility of skin lesions may further lead to social isolation [10]. Recognizing these challenges, the current psychosocial care guidelines for EB emphasize improving social participation as a key priority [11].

Evidence from diverse disability populations shows that participation outcomes are shaped by both environmental and personal factors [12, 13]. Environmental barriers include limited accessibility, stigma, and insufficient policy support, whereas supportive relationships and inclusive social environments often serve as facilitators [12–14]. However, studies across different disability groups indicate that environmental and personal factors influence participation in distinct ways across disease populations, underscoring the need for condition-specific investigations [15].

To date, participation of EB patients has predominantly been addressed in the paediatric population and their involvement in school [6, 16, 17]. Participation in education may be restricted by stigmatization, frequent absences, and peers’ misconceptions about EB [6, 17]. The importance of school participation is well established and reflected in the informational resources provided by patient organizations and healthcare facilities^1^. However, participation in adulthood remains an underexplored aspect of living with EB. Over 70% of adults report that EB affects their career choices or employment opportunities, yet the personal implications of these challenges have not been examined in depth [18, 19]. Moreover, EB places a considerable financial burden on patients, with out-of-pocket costs estimated at approximately €4,129 per year [20]. Hence, this study explores the social participation of EB patients in three major life areas: (a) education, (b) work and employment, and (c) economic life [21].

The International Classification of Functioning, Disability and Health (ICF) provides the theoretical framework for this study, conceptualizing participation as an individual’s “involvement in a life situation” [21]. More recent approaches have moved toward a more nuanced understanding of participation that includes both observable behaviour (e.g., frequency of activity or attendance) and the individual’s subjective appraisal of involvement [22, 23]. Aligned with this perspective, participation in the present study is conceptualized not only as physical presence but also as the individual’s perception of involvement [24]. Furthermore, the ICF emphasizes the interaction between a person’s health condition and environmental factors, which can either facilitate or hinder participation [21].

In line with this framework, this study examines barriers and facilitators to the social participation of individuals with EB in education, work and employment, and economic life, thereby addressing critical but underexplored domains of adult participation.

## Methods

A qualitative research design was employed. Biographic interviews were analysed according to Mayring´s qualitative content analysis [25], informed by a critical realist ontological position [26]. The ‘Consolidated Criteria for Reporting Qualitative Research’ (COREQ) were used to ensure methodological transparency and rigor [27].

### Participants and recruitment

Participants were recruited at the EB centre in Freiburg, Germany. Purposive sampling ensured diverse representations across EB types, disease severity levels, ages, and genders. Eligible participants were required to have a confirmed diagnosis of EB, be at least 18 years old, reside in Germany, and be able to communicate verbally in German to provide biographical narrations. Recruitment and interviews took place between August 2024 and January 2025. Clinicians contacted nineteen patients. The first participant was selected based on availability and willingness to participate, meeting the inclusion criteria. Five patients either did not reply or declined participation, and two interviews were cancelled for scheduling reasons or non-responsiveness. Thus, twelve interviews were ultimately conducted. Of the seven individuals who did not participate, two had EBS, three had DEB, and two had JEB, two were male and five were female. Although additional interviews were planned, recruitment was limited by the rarity of EB and the small eligible patient population. Nevertheless, the sample reflected sufficient variation in EB type, disease severity, age, and gender to capture a wide range of perspectives.

### Study setting and data collection

The interviews were conducted by a psychologist (VH) with experience in qualitative interviewing. They took place either in person at the clinic facilities, via telephone, or through video calls, depending on the participants’ preferences. The interview guide was reviewed by two researchers and one clinician experienced with EB prior to data collection. Minor adjustments were made after the first interview, which served as a pilot to improve question clarity. All interviews, including the pilot, were included in the final analysis. The interviewer was acquainted with two participants through prior encounters at patient organization meetings but had no personal or therapeutic relationships with them.

A semi-structured biographical interview approach was used to explore how participants’ experiences of participation in education, employment, and economic life unfolded over time. This approach enabled participants to situate their experiences within a chronological life course and to link key transitions and changing environmental factors to participation. The interviews followed a three-part structure. First, the participants were invited to give a broad account of their life history from childhood and school to the present. The second part focused on significant events and experiences related to social participation, including barriers and facilitators in education, work, and economic life. This section aimed to gather insights into the importance participants attributed to specific life areas, as well as their subjective sense of involvement, which are both constituting factors of participation. The final section included the collection of sociodemographic data (see complete interview guide in Additional File 1). Field notes were taken during the interviews to document contextual observations, support the formulation of follow-up questions, and inform the subsequent data analysis.

All interviews were audio-recorded and transcribed verbatim. The average interview duration was 95 min (range: 62–185 min). Interview transcripts were not returned to participants for comment or correction, and the results were not shared. However, preliminary findings were presented and discussed with patients at a patient organization meeting, and the feedback obtained informed the final interpretation of the results.

### Data analysis

The interviews were analysed using a deductive coding system, according to qualitative content analysis [25]. Coding was primarily conducted by one researcher (VH). To enhance reliability, a second researcher (LB) independently coded two transcripts to verify the coding system’s applicability, and discrepancies were resolved through discussion and consensus.

The coding system was based on the ICF components *Activities and Participation* and *Environmental Factors*, which form the two main components of the analysis (Table 1). The participation codes were derived from the ICF chapter ‘Major Life Areas’. The codes covering environmental factors encompassed the full set of barriers and facilitators outlined in the ICF, except for ‘Natural Environment and Human-Made Changes to Environment’, which was excluded due to limited relevance in the interviews. As the coding system was derived directly from the established ICF framework, no inductive codes were constructed to ensure that the analysis remained conceptually anchored in the established classification system. Text segments that did not fit existing categories were coded under an “Other” category to ensure that relevant content outside the predefined structure was retained for interpretive consideration. Although a formal saturation analysis was not performed, emerging themes were monitored throughout coding, and later interviews did not yield substantially new themes.

Data analysis was performed via the MAXQDA software [28]. All codes were applied in either a positive sense, indicating participation or facilitator, or a negative sense, indicating participation restriction or barrier. Disease severity was also considered: a patient was considered severely affected if restricted hand function or the use of mobility aids was reported. In the context of social participation, these factors serve as meaningful proxies for disease severity, as they directly impact an individual’s ability to engage in daily activities and access public spaces [29, 30].

**Table 1:** Structure of the coding system. (adapted from ICF [21])

Components	Domains^a^	Definitions^b^
Participation Participation Participation restriction	Major Life Areas Education Work and Employment Economic Life	Being involved in education, work, and employment, and having command over economic resources.
Environmental Factors Barriers Facilitators	Products and Technology	Product, instrument, equipment or technology adapted or specially designed for improving the functioning.
	Support and Relationships	People that provide practical physical or emotional support, nurturing, protection, assistance.
	Attitudes	Attitudes that are the observable consequences of customs, practices, ideologies, values, norms, factual beliefs and religious beliefs.
	Services, systems, and policies	Services that provide benefits and structured programmes. Systems that are administrative, control and organizational mechanisms. Policies constituted by rules, regulations, conventions and standards established by governments at the local, regional, national, and international levels.

^a^ The exact codes included in the respective domains can be retrieved from the ICF. Codes down to the four-digit level have been included (e.g., e310 - support of immediate family)

^b^ Definitions have been adapted from ICF

### Ethical considerations

Ethical approval for this study was obtained from the ethics committee of Albert-Ludwigs-University Freiburg (24-1147-S1). All participants provided written informed consent before the interviews were conducted and the interview transcripts were pseudonymized before analysis. All audio recordings, verbatim transcripts, and analysis materials were stored on encrypted institutional servers accessible only to the research team. Audio recordings were deleted after verbatim transcription.

## Results

A total of twelve interviews were conducted. The sample characteristics are shown in Table 2. The average age of the participants was 37.7 years (range 20–70 years). Each DEB (*n* = 5) and JEB (*n* = 5) account for 41.66% of the sample. Two EBS cases account for the remaining 16.66%. Disease severity, in this study defined as restricted hand function and/or the use of mobility aids, was evenly distributed: 50% moderate (*n* = 6) and 50% severe (*n* = 6).

**Table 2:** Sociodemographic characteristics

	*n*	%
Sex		
Female	6	50
Male	6	50
EB Type		
EBS	2	16.67
DEB	5	41.67
JEB	5	41.67
Age		
18–29 years	4	33.33
30–39 years	5	41.67
40–49 years	1	8.33
≥ 50 years	2	16.67
Employment Status		
Employed	7	58.33
Not employed	3	25
In education	2	16.67
Hand Function		
Hand function restricted	5	41.67
Hand function is not restricted	7	58.33
Mobility Aids		
Used	4	33.3
Not used	8	66.7

Altogether, 17 environmental factors that impact participation in the investigated life areas were identified. A summary of the identified barriers and facilitators is provided in Table 3, along with example quotes^2^. Throughout the analysis, education was further divided into ‘school education’ and ‘higher education and vocational training’ as a shift in the associated environmental factors towards the later stages of education became evident.

**Table 3:** Identified barriers and facilitators to participation in education, work and employment and economic life

	Environmental Factors	
	Barrier	Facilitator
**Education**		
School education	**Peers attitudes**: “I remember one time I sat down next to someone, and he immediately turned away from me, like pushed himself away, and then said to me, ‘Please do not touch me’” (Interview 8, Pos. 49)	**Supportive parents**: “[…] my mom made sure that I attended school, no matter what” (Interview 6, Pos. 4). **Supportive teachers/educational institution**: “Fortunately, I had a principal and teachers who were understanding and gave me some leeway” (Interview 5, Pos. 24). **Supportive peers**: “There was always someone driving, and my friends were also in the same extracurricular groups, so we could get there and back together. And little things like that are actually big factors in whether I can actively participate or not” (Interview 4, Pos. 37).
Higher education and vocational training	**Attitudes of employers (vocational training)**: “[…] No one wanted to take me. Because of my condition, I wasn’t accepted into the program” (Interview 3, Pos. 9). **Labour and employment services**: “I would have wished for more [from employment agency] help in finding something new or perhaps sitting down with me to see what else I could do” (Interview 12, Pos. 6). **Non-supportive educational staff/institution**: “I had to fight really hard to be allowed to take my exam without handwriting” (Interview 4, Pos. 30).	**Supportive educational staff and institutions**: “Our school director was a progressive thinker and said, ‘This won’t be easy for you, but let’s try it together’” (Interview 9, Pos. 15). **Educational policies**: “During my studies, I was able to apply for a so-called ‘medical leave semester.’ […] That extra time made it possible for me to complete my first state exam successfully” (Interview 1, Pos. 9).
**Work and Employment**	**Labour and employment services**: “I thought, ‘They’re supposed to help me, not judge me,’ but the advisor just looked at me harshly when I explained my situation” (Interview 2, Pos. 11). **Attitudes of employers**: “As soon as they saw ‘physically disabled,’ they thought, ‘We do not need that’. When they saw me in person, they just threw up their hands in despair” (Interview 3, Pos. 21). **Attitudes of colleagues**: “In those jobs, you need a clear head and must analyse things rather than just focusing on your own fate, which I think I do quite well. But instead, my colleagues tend to assume that I am too biased” (Interview 9, Pos. 17).	**Supportive colleagues**: “Every two or three months, I would just be out for two, three, or four days because of my esophagus, and it was never really a topic. It was just like, ‘All the best, take care, we wish you luck’” (Interview 7, Pos. 32). **Supportive employers** (**Flexible working hours and conditions)**: “I can do my job from my home office, which is super relaxing. I wear sweatpants and flip-flops—or sometimes no shoes at all which really helps with my affected skin areas” (Interview 11, Pos. 28).
**Economic Life**	**Social security system**: “Then it happens that you submit something, and then it gets rejected outright, so you have to file an appeal. Then the appeal gets rejected. And then you just give up” (Interview 12, Pos. 36). **Health insurance systems**: “The fight with the health insurance companies is enormous” (Interview 5, Pos. 91).	**Patient organizations**: “The patient organization is really, really good. EB is a priority for them, and if something needs to be handled, like paperwork, which there is a lot of. […] They take care of it.” (Interview 6, Pos. 18).

### Education

#### Participation in school

Collaborative efforts among teachers, parents, and peers led to the creation of an inclusive school environment. Furthermore, the stability of the school environment provided a foundation for sustaining special adaptations long-term, which enabled consistent school attendance for the student with EB. However, restrictions on participation in the social sphere of school were also evident.

#### Barriers to participation in school

Inappropriate comments, bullying, or social exclusion by **peers** were experienced by all participants at least once and were the most evident barriers to participation in school. Negative reactions from peers stemmed from visible differences (e.g. visible wounds or missing fingernails) or misconceptions such as EB being a contagious disease. The participants shared similar experiences, one example being *“So*,* they distanced themselves from me*,* keeping their distance in a way that I was sometimes completely alone at school”* (Interview 8, Pos. 49; JEB, female, restricted hand function, no mobility aids). In addition to physical differences, participants reported that peers were resentful or unaccepting when special arrangements were made, such as allowances for more frequent absences.

#### Facilitators to participation in school

**Family support** emerged as a factor in promoting school participation. Parents often actively advocated for their children, ensuring that accommodations were in place. This included ensuring that schools could meet the child´s needs even before school enrolment, whether by assessing accessibility, school, and class size (as crowded schools and playgrounds were perceived as a potential threat), opportunities for active involvement, or opting for a school specifically for students with physical disabilities. These measures helped ensure that participants were able to attend school. As one participant shared, *“My mom made sure that I attended school*,* no matter what*,* so that I could develop some kind of social contacts. […] She managed to do this by applying for a position as a librarian at the school’s library”* (Interview 6, Pos. 4; DEB, male, restricted hand function, mobility aids used).

**Supportive teachers** and school institutions contributed to the creation of a protective and simultaneously inclusive environment. For example, some educators ensured that adjustments were in place that facilitated peer interactions while also mitigating risks: *“I could get bumped into or jostled*,* and the playground could be quite wild […]. So*,* instead*,* I was allowed to stay inside and also to bring my friends”* (Interview 10, Pos. 14; JEB, female, hand function not restricted, no mobility aids).

While peers act as the most frequent barrier to participation, they are also crucial facilitators in enhancing school participation. Although special adjustments, such as separate break spaces, were sometimes necessary to ensure safety, they also had the potential to create unintended social exclusion. Supportive peers helped mitigate these effects by accompanying students with EB, thereby reducing the isolating effects of such measures.

#### Participation in higher education and vocational training

In the later stages of education, participants made strategic educational choices. Most participants chose their educational paths based on feasibility, not always on personal interest, opting for fields with minimal physical strain or that allowed remote work: *“I chose a field where I knew I would have good chances of working completely remotely in the future because my physical condition wasn’t improving”* (Interview 4, Pos. 6; EBS, female, hand function not restricted, mobility aids used).

In addition, the circumstances in higher education and vocational training were described as less stable. Frequent transitions in educational staff, classrooms, and peers required participants to continuously renegotiate special arrangements, which used to be a one-time task. On the other hand, bullying and inappropriate comments, the key barriers in school education, became less prevalent as peers matured. However, overall interaction with peers and joining extracurricular activities decreased for some participants as opportunities to engage in student life became less accessible: *“During my school years*,* I was very active*,* and I really enjoyed that. But in university*,* things changed. I wanted to be more involved*,* but there were just no opportunities that fit my condition”* (Interview 4, Pos. 53; EBS, female, hand function not restricted, mobility aids used). Individuals with moderate forms of EB experienced fewer difficulties in both the participation and completion of their education.

#### Barriers to participating in higher education and vocational training

Severely affected participants faced challenges in securing vocational training placements, often perceiving rejections as discrimination (coded as **attitudes of employers** (vocational training)). One participant recounted, “*I actually wanted to become a tax clerk*,* but because of my illness*,* no one wanted to take me. Because of my condition*,* I wasn’t accepted into the program*” (Interview 3, Pos. 9; DEB, male, restricted hand function, mobility aids used). In contrast, enrolment in higher education was described as less burdensome due to standardized admission procedures. Additionally, supportive policies^3^ enabled students with EB to enrol in the desired programs.

Since securing vocational training placement was particularly challenging for severely affected participants, interactions with **labour and employment services** were frequently discussed. While these agencies are intended to facilitate vocational training, participants found the available options poorly suited to their (social) needs: “*The only other option they offered me was an online training program. […] that wasn’t an option for me*,* I want to work with people*,* not just sit at home*” (Interview 12, Pos. 6; DEB, male, restricted hand function, mobility aids used).

In narratives on education, **non-supportive educational staff** and institutions were identified as barriers. The participants described educational environments as inflexible and unprepared to meet their needs. In one case, obtaining approval for necessary exam accommodations was described as a struggle, whereas this had not been the case in school education: “*I had to fight really hard to be allowed to take my exam without handwriting. I had bandages and blisters on my fingers and needed more time to write*” (Interview 4, Pos. 30; EBS, female, hand function not restricted, mobility aids used).

#### Facilitators to participation in higher education and vocational training

Others reported that **supportive educational staff** and supervisors enhanced their academic experiences. The presence of understanding and proactive educators who were willing to address and mitigate challenges associated with their condition served as a facilitator: “*Our school director was a progressive thinker and said*,* ‘This will not be easy for you*,* but let’s try it together*’” (Interview 9, Pos. 15; DEB, male, restricted hand function, no mobility aids).

In addition, certain **educational policies** played a critical role in facilitating participation in higher education and vocational training. Policies allowing for extended study periods or medical leave enabled participants to balance academic responsibilities with health needs. These policies fostered equitable opportunities. One participant highlighted the significance of one specific policy: “*During my studies*,* I was able to apply for a so-called ‘medical leave semester.’ […] That extra time made it possible for me to complete my first state exam successfully*” (Interview 1, Pos. 9; JEB, female, hand function not restricted, no mobility aids).

#### Participation in work and employment

Finding employment was frequently perceived as a challenge for those with severe EB, even when feasible professions were pursued. However, once employed, workplace adaptations facilitated participation. Those with milder EB reported fewer restrictions, benefiting from supportive colleagues and employers. Beyond financial independence, employment also mitigated social isolation. One participant reflected, “*Why working is so good for you is because you escape*,* you break out of this prison and you simply experience something different. You meet people who do not make you feel like you are a burden*” (Interview 6, Pos. 10; DEB, male, restricted hand function, mobility aids used).

#### Barriers to participation in work and employment

Participants with severe EB frequently faced discrimination in the hiring process, encountering biases related to their disability and appearance (categorized as **attitudes of employers**). Many expressed frustrations, as they were overlooked for positions based on appearance and disability. As one participant recounted, “*As soon as they saw ‘physically disabled’*,* they thought*,* ‘We do not need that’*,* when they saw me in person*,* they just threw up their hands in despair*” (Interview 3, Pos. 21; DEB, male, restricted hand function, mobility aids used). Another participant shared, “*I wanted to work*,* but society did not accept me. I tried so much*,* but after two years*,* I still couldn’t find a job*” (Interview 2, Pos. 11, JEB, male, restricted hand function, no mobility aids).

A further challenge stemmed from the perceived lack of support from **labour and employment services**. The participants described the employment agencies as ill-informed about EB and the respective restrictions and possibilities, resulting in unrealistic job placements. One participant recounted being assigned a job he is physically not able to do: *“I was sent here to the clinic*,* and I was supposed to carry files from point A to point B in the basement*,* which I cannot do at all because of my EB. They had no idea what to do with me”* (Interview 3, Pos. 17; male, DEB, restricted hand function, mobility aids used).

Additionally, workplace **colleagues** misjudged participants’ (mental) abilities. In some cases, assumptions about their limitations overshadowed their professional competencies. One participant working with vulnerable groups explained: *“In those jobs*,* you need a clear head and must analyse things rather than just focusing on your own fate*,* which I think I do quite well. But instead*,* my colleagues tend to assume that I am too biased”* (Interview 9, Pos. 17; JEB, male, restricted hand function, no mobility aids).

#### Facilitators to participation in work and employment

One key facilitator was the presence of **supportive colleagues**. Participants felt valued when their disability was acknowledged without being the focal point of workplace interactions. One participant said: “*My colleagues don’t see me as someone with a disability but as a normal employee. When I need help*,* they offer it without making me feel dependent*” (Interview 6, Pos. 8; DEB, male, restricted hand function, mobility aids used).

**Supportive employers** who were willing to provide flexibility in working hours and conditions were another facilitator. Adjustable schedules and part-time positions allowed participants to manage their EB while maintaining employment. In addition, remote work opportunities facilitated employment, by minimizing physical strain, as one participant shared: *“I can do my job from my home office*,* which is super relaxing. I wear sweatpants and flip-flops—or sometimes no shoes at all*,* which really helps with my affected skin areas”* (Interview 11, Pos. 28; JEB, male, hand function not restricted, no mobility aids).

### Economic life

Participants relied on multiple financial resources, including reimbursements for medical supplies, travel costs to EB specialists, mobility aids, and social security benefits. However, these resources often functioned more as barriers than facilitators due to the high administrative burden. Many individuals struggled to obtain the benefits they believed they were entitled to, highlighting not only financial strain but also significant psychological stress. Participants perceived the available support as insufficient, difficult to access, or unfairly denied. For this reason, social security and health insurance services are discussed as barriers. In contrast, support from patient organizations emerged as a key facilitator, as they frequently assisted with bureaucratic challenges.

#### Barriers to participation in economic life

A major challenge for participants was the complex and time-consuming process of dealing with **social security services** to secure financial support and disability benefits. Navigating administrative systems required significant effort and often adds to the difficulties of daily life. This administrative burden was often exacerbated by the need for repeated justification of one’s condition, as one participant expressed: *“These were often the things that stressed me the most. I kept thinking*,* ‘Why don’t they believe me?’ Now I have to write 30 letters*,* run to the doctor again*,* get another certificate*,* and even then […]. It drains your energy”* (Interview 9, Pos. 19; JEB, male, hand function restricted, no mobility aids).

Participants often had similar experiences with **health insurance services** and struggled with high out-of-pocket expenses for medical supplies. For some, these additional costs meant that they had to make sacrifices elsewhere, for example, limiting their use of high-quality bandages or medical products to the most essential situations. One participant highlighted the financial strain of obtaining necessary wound care products: “*I also do less than I should when it comes to wound care because getting the materials is such a hassle. It is expensive. Sure*,* a lot is covered*,* but if I buy a pack of [wound care product]*,* I still have to pay 8 euros. If I would use it on every wound*,* it would not fit within my budget*” (Interview 5, Pos. 91; DEB, female, hand function not restricted, no mobility aids).

#### Facilitators to participation in economic life

The most substantial facilitator was support from patient organizations. These organizations provided essential guidance in managing complex administrative processes and accessing resources. Their assistance was particularly valuable for individuals struggling with formal paperwork. As one participant explained, “*The patient organization is really*,* really good. EB is a priority for them*,* and if something needs to be handled*,* like paperwork*,* which there is a lot of. […] They take care of it*” (Interview 6, Pos. 18; DEB, male, restricted hand function, mobility aids used). This external support considerably reduced the burden of navigating social support and medical systems.

## Discussion

This study explored the barriers and facilitators affecting the social participation of individuals with EB in education, employment, and economic life. Biographic interviews provided insights into experiences across different life stages. The findings highlight and describe 17 environmental factors.

Bullying and social exclusion emerged as major issues in school settings. Aligning with Salamon et al., our results suggest that a supportive environment, including family, educators, and peers can mitigate social challenges [5]. To prevent problems, parents ensured that schools were willing and able to make concessions even before school enrolment. Educators played a key role in providing inclusive learning environments that simultaneously safeguard but do not exclude students with EB. The solidarity of peers, who accompanied students with EB in situations in which special adjustments had adverse effects (e.g., separate break spaces), reduced the burden of not being able to attend certain activities. This highlights the need for a balanced approach, where protective measures do not unintentionally exclude individuals from social interaction. During primary and secondary education, students with EB benefited from a stable environment where individualized solutions, once established, can be consistently maintained.

In higher education, frequent changes in teaching staff meant that students had to repeatedly adapt and renegotiate special adjustments. The difficulties in adjusting to higher education are in line with previous studies that examined the participation of people with disabilities in higher education [31, 32]. Notably, access to higher education was less challenging due to standardized enrollment procedures in Germany. In contrast, access to vocational training was perceived as more challenging. Additionally, multiple participants struggled to complete their education. Two key factors linked to dropout among students with disabilities, social integration and financial stability, were also evident in participants’ narratives [33]. Although explicit social exclusion and negative verbal comments were less frequent as peers matured, the activities of the student life were often inaccessible, resulting in fewer social contacts.

Concerning participation in work, finding an employer willing to accommodate a person with EB was particularly difficult. Research on employment and EB is limited, but existing studies on the overall impact of EB highlight struggles in securing employment and the impact on career choices [18, 19]. The participants in this study described hiring practices as discriminatory, which aligns with broader evidence on the employment of people with disabilities [34, 35]. However, once employed, most participants experienced support from colleagues. Additionally, remote work possibilities acted as facilitators. Previous studies have referred to remote work as a “silver lining” of the COVID-19 pandemic for people with disabilities [36]. The perceptions of remote opportunities varied among the participants, while some valued the recovery it provided, other participants felt that it would isolate and disconnect them from social life. This reflects a broader paradox regarding special accommodations: while certain adjustments may improve accessibility, they may also limit social interactions [37]. In summary, difficulties in securing a position hinder participation in vocational training and employment, as access is a prerequisite for participation. In contrast, when participation in school and higher education was restricted, it was primarily due to difficulties integrating with peers, impacting the individual’s sense of belonging.

The economic burden of EB and the high associated out-of-pocket expenses have been previously quantified [20]. Economic life can be understood both as a domain of participation in its own right and as an enabler of participation in other areas. This study highlights the struggles in accessing reimbursements for medical supplies, assistive devices, and social security benefits. The participants described the bureaucratic process as emotionally exhausting and time-consuming, and even abandoning or withdrawing applications despite being eligible. This reflects the phenomenon of “non-take-up” of benefits, where people do not claim social security support due to complex procedures and administrative hurdles [38]. The role of patient organizations in reducing bureaucratic stress was also emphasized, underscoring the importance of support systems in navigating administrative challenges. The results also revealed paradoxical environmental factors, such as support systems (e.g., social security), becoming barriers due to their high accessibility thresholds. The ICF framework helped identify these effects by emphasizing that an environmental factor can act as both a barrier and facilitator [21].

Taken together, these findings are consistent with broader disability research indicating that participation is context-dependent and shaped by social, institutional, and attitudinal environments [14, 15, 21, 39]. This study extends that understanding by highlighting the paradoxical nature of certain adaptations for individuals with EB. Measures intended to support patients, such as remote work, or protective measures may inadvertently reinforce social separation. This underscores that interventions intended to facilitate participation may, in some cases, have unintended adverse effects. Therefore, measures to enhance participation among individuals with EB should carefully consider potential unintended consequences.

### Strengths and limitations

By capturing personal experiences across different life stages, this study provides a comprehensive understanding of the identified barriers and facilitators in education, employment, and economic life. The application of the ICF framework ensured a structured analysis. This study also contributes to relatively underexplored areas, particularly regarding employment and economic life.

As coding was conducted primarily by one researcher, the reliability of the coding process could not be formally verified. However, adherence to a structured, theory-based coding framework helped maintain analytical consistency. Furthermore, the use of a deductive coding approach inherently limited the identification of themes not encompassed within the ICF. Although later interviews did not yield substantially new themes, data saturation was not formally assessed, and additional perspectives may therefore remain unrepresented. Furthermore, the findings are largely restricted to the German context. Since environmental factors differ across countries, the findings may not be fully generalizable to other contexts. Additionally, personal factors, such as coping strategies, psychological traits, or socioeconomic status, were not explored in depth. According to the ICF model, personal factors are a crucial component for understanding functioning (including participation). Future research should examine the role of personal factors more closely, thereby contributing to a more comprehensive application of the ICF model to EB.

### Implications

In the German context, it is important to acknowledge that participation is legally anchored through the ‘Federal Participation Act’ [40]. However, a persistent gap between legal entitlement and lived experience remains [41]. The findings of this study suggest two potential avenues for addressing this gap. First, the high threshold for accessing social support systems can be directly addressed. Patients would highly benefit from skilled support (e.g., by social workers familiar with EB). Professional support could help identify the available resources, lessen the burden of accessing them, and prevent non-take-up of benefits. However, ensuring equitable access to social security services requires the formal recognition of rare conditions such as EB within administrative frameworks.

Second, the attitudes of uninformed individuals toward the participants complicated their ability to find employment, get special accommodations approved, and access public resources. Moreover, such attitudes restrict not only access but also involvement by potentially undermining the individual’s sense of belonging. There is plausible evidence that awareness campaigns can reduce bias [42]. Supporting patient organizations in their existing efforts to raise awareness about EB may help reduce stigma, which, consequently, may facilitate participation in all social fields.

In addition to practical implications, the findings suggest several directions for future research. First, while much existing research has focused on children and adolescents with EB [6, 16, 17], the present study highlights psychosocial challenges faced by adults, particularly regarding participation in employment and economic life, which warrant further investigation. Second, personal factors, an important influence on participation processes [21], were not explicitly considered in this study and should be integrated in future research to provide a more comprehensive understanding of participation outcomes in EB [13, 43].

## Conclusion

Living with EB often means being careful and aware of stressors on the skin, but protective measures can inadvertently limit physical activity and overall participation. This dilemma highlights the need to address the social participation of EB patients. This study has taken a first exploratory step by identifying environmental factors contributing to participation across education, work and employment, and economic life. Multiple facilitators mitigated barriers to participation in school. In the later stages of education and employment, the identified facilitators became less effective, often resulting in a low degree of participation. In addition, this study emphasizes that certain adjustments (e.g., separate break spaces in school or remote work) can have adverse effects. These possible unintended effects must be considered when implementing solutions for participation. In terms of economic participation, the high threshold for accessing public resources and reimbursement for medical supplies must be noted. Addressing the accessibility of social support systems and increasing public awareness of EB through education and advocacy efforts could enhance the participation and overall well-being of individuals living with EB.

## Electronic Supplementary Material

Below is the link to the electronic supplementary material.

Supplementary Material 1

## Data Availability

The data generated and analysed during the current study are not publicly available due to the biographical nature of the interviews, as participants’ personal information may not be fully pseudonymized.

## Footnotes

1 For the German context most notably the DEBRA patientorganizations and specialized EB centers provide informational materials to facilitate school inclusion.

2 Patient quotes have been translated from German to English by the authors.

3 In Germany, applicants with disabilities may be granted equity measures in university admissions to ensure fair access,including adjusted entry thresholds.

## Abbreviations

EB: Epidermolysis bullosa
ICF: International Classification of Functioning, Disability and Health
