# Supplementary material for: Social participation of patients with epidermolysis bullosa: barriers and facilitators in education, employment, and economic life - a biographic interview study
Source: Orphanet J Rare Dis. 2026 Mar 25;21:138. doi: 10.1186/s13023-026-04329-y (PMC13063464; doi:10.1186/s13023-026-04329-y)
Supplement: Supplementary file 1 — Supplementary Material 1 [file 13023_2026_4329_MOESM1_ESM.docx]

**Interview Guide**:

1. **“To begin, I would like to invite you to tell me about your life, starting from your early childhood and school years continuing up to the present day.**”
   1. Immanent follow-up questions
2. **“Now that you have shared an overview of your life history, I would like to invite you to reflect on specific events or periods that you consider significant, particularly in relation to participation.**”
   1. Immanent follow-up questions
3. “Are there any topics or aspects that we have not discussed, but that you consider important?”
4. Sociodemographic information:
   1. Age
   2. Gender
   3. EB type
   4. Occupation
   5. Restricted hand function (Yes/No)
   6. Use of mobility aids (Yes/No)
